# Droplet digital PCR-based circulating microRNA detection serve as a promising diagnostic method for gastric cancer

**DOI:** 10.1186/s12885-018-4601-5

**Published:** 2018-06-22

**Authors:** Gaoping Zhao, Tao Jiang, Yanzhuo Liu, Guoli Huai, Chunbin Lan, Guiquan Li, Guiqing Jia, Kang Wang, Maozhu Yang

**Affiliations:** 1 Department of Gastrointestinal Surgery, Sichuan Academy of Medical Sciences & Sichuan Provincial People’s Hospital, Chengdu, 610072 China; 2 School of Medicine, University of Electronic Science and Technology of China, Chengdu, 610054 China; 3 Institute of Chengdu Biology, and Sichuan Translational Medicine Hospital, Chinese Academy of Sciences, Chengdu, 610041 China; 4 Department of General Surgery, Qionglai Medical Center Hospital, Chengdu, Sichuan Province, China, Chengdu, 611530 China; 5 Department of General Surgery, Sichuan Academy of Medical Sciences & Sichuan Provincial People’s Hospital, Chengdu, 610072 China

**Keywords:** Gastric cancer, Liquid biopsy, ddPCR, miR-21, miR-93, miR-106a, miR-106b

## Abstract

**Background:**

Novel non-invasive biomarkers for gastric cancer (GC) are needed, because the present diagnostic methods for GC are either invasive or insensitive and non-specific in clinic. The presence of stable circulating microRNAs (miRNAs) in plasma suggested a promising role as GC biomarkers.

**Methods:**

Based on the quantitative droplet digital PCR (ddPCR), four miRNAs (miR-21, miR-93, miR-106a and miR-106b) related to the presence of GC were identified in plasma from a training cohort of 147 participants and a validation cohort of 28 participants.

**Results:**

All circulating miRNA levels were significantly higher in the plasma of GC patients compared to healthy controls (*P* < 0.05). Through a combination of four miRNAs by logistic regression model, receiver operating characteristic (ROC) analyses yielded the highest AUC value of 0.887 in discriminating GC patients from healthy volunteers. Furthermore, miR-21, miR-93 and miR-106b levels were significantly related to an advanced TNM stage in GC patients. ROC analyses of the combined miRNA panel also showed the highest AUC value of 0.809 in discriminating GC patients with TNM stage I and II from stage III and IV. Through combining four miRNAs and clinical parameters, a classical random forest model was established in the training stage. In the validation cohort, it correctly discriminated 23 out of 28 samples in the blinded phase (false rate, 17.8%).

**Conclusions:**

Using the ddPCR technique, circulating miR-21, miR-93, miR-106a and miR-106b could be used as diagnostic plasma biomarkers in gastric cancer patients.

**Electronic supplementary material:**

The online version of this article (10.1186/s12885-018-4601-5) contains supplementary material, which is available to authorized users.

## Background

Gastric cancer is second most common cancer in terms of incidence and mortality in China, according to the most recent cancer statistics [1]. With the improvement of surgical technique, radiotherapy and chemotherapy in recent years, patients in the early stage of GC had a significant increased 5-year survival rate, but the prognosis for advanced GC remains poor [2, 3]. Thus, it is important to diagnose GC in the early stage thus yielding better outcome. Gastroscopy is the gold standard test for GC diagnosis, but it is invasive and couldn’t be frequently used as regular health examination. Carcinoembryonic antigen (CEA), α-fetoprotein (AFP) and carbohydrate antigen 19–9 (CA19–9) are widely used as non-invasive markers in clinical, but their sensitivities and specificities are not enough for early diagnosis of GC [4]. Therefore, novel non-invasive biomarkers with better sensitivities and specificities are urgently needed.

MicroRNAs (miRNAs) are small noncoding RNAs, about 22–24 bases long, that inhibit their target mRNAs translation by inducing mRNA degradation or translational repression [5, 6]. Up to now, there are thousands of miRNAs have been reported to be associated with tumor growth, invasion, metastasis and apoptosis [7]. Several studies have demonstrated that circulating miRNAs can serve as biomarkers for GC diagnosis. For example, miR-223, miR-16 and miR-100 were highly expressed in the serum of GC patients, and positively associated with TNM stage, metastatic status, tumor size and differentiation grade [8]. The level of let-7a expression in the plasma of GC patients was significant lower, and the value of the area under the receiver-operating characteristic curve was 0.879 for the miR-106a/let-7a ratio in GC patients and healthy volunteers [9]. Thus, miRNAs in peripheral blood have great potential for helping early diagnosis of GC.

Although the results of previous studies are promising, their clinical transferability remains uncertain, which mainly due to the lack of uniformity and reproducibility in the criteria for determining the circulating miRNA levels by quantitative real-time PCR (qPCR). Besides, several variables such as sample storage, RNA isolation, PCR inhibitors and normalization could affect final results [10]. The droplet digital PCR (ddPCR) technique is increasingly considered to be the gold standard in the application of liquid biopsy, because it has shown superior precision and sensitivity, being less affected by PCR inhibitors, and unnecessity of internal/external normalization while detecting low concentration of target nucleic acids molecules [11, 12]. In this study, we used the ddPCR technique to explore the circulating miRNA signatures which could be potential biomarkers for GC diagnosis, and discriminating GC patients with different TNM stage. Four miRNAs, miR-21, miR-93, miR-106a and miR-106b, which have been **most reported** to be closely correlated with GC in tissue and plasma of patients and represent as candidate biomarkers for human GC, were examined by novel technique of ddPCR. [9, 13–15].

Furthermore, without the assist of tissue biopsy and imaging examinations, it would be difficult for clinicians to make diagnosis and tumor staging for GC, because there are many factors could probably influence the results. To improve the precision and accuracy of diagnosing disease, new approaches such as machine learning which is the main technical basis for data mining, provide an effective solution [16]. Several studies have been reported to use machine learning tools for data mining to diagnose disease or predict prognosis [17–19]. In this study, we explored the use of random forest model based learning for GC diagnosis, by using circulating miRNA expressions and clinical parameters such as age, gender, CEA and CA19–9.

## Methods

### Patients and blood samples

The present study was approved by the ethics committee of Sichuan Provincial People’s Hospital. All participants provided written informed consent form to approve the use of their blood samples for research purposes.

From Sichuan Provincial People’s Hospital, a total of 101 patients with gastric cancer (GC) and 46 healthy volunteers were recruited to the training cohort between January 2017 and June 2017, and a total of 11 patients with GC and 17 healthy volunteers were recruited to the validation cohort between December 2017 and February 2018. For plasma, 5 ml peripheral blood was collected in EDTA tubes, the sampling time was pre-surgery for GC patients, especially. And within 2 h, plasma was separated by centrifugation at 2000×g for 10 min, the supernatant was followed by a second centrifugation at 12000×g for 20 min. Then, the plasma was either stored at − 80 °C or miRNA was extracted immediately.

For patients, GC paraffin-embedded tissue samples were obtained after surgical resection. The clinicopathological classification and staging were determined according to the World Health Organization pathological classification of tumors. The clinical information for GC patients in the training stage is summarized in Table 1. Among the 101 patients included 51 male and 50 female, the median age was 56 years old (range, 35–75 years) and the median tumor size was 3.9 cm (range, 1.0–7.5 cm). There were 16 cases well differentiated, 35 were moderately differentiated and 50 were poorly differentiated. There were 35 cases without lymph node metastasis, 66 cases with lymph node metastasis, 18 cases with distant metastasis and 83 cases without distant metastasis. According to TNM stage classification, 28 cases were categorized as stage I, 13 cases for stage II, 36 cases for stage III and 24 cases for stage IV.

**Table 1:** Clinicopathological characteristics of all individuals in the training stage and relationships with circulating miRNAs in the plasma

Characteristics		miR-21		miR-93		miR-106a		miR-106b	
	Total (%)	Mean ± SD	*P* value	Mean ± SD	*P* value	Mean ± SD	*P* value	Mean ± SD	*P* value
GC patients									
Gender									
Male	51(50.5)	300.2 ± 127.7	0.437	206.3 ± 87.8	0.318	41.3 ± 18.7	0.404	26.3 ± 11.8	0.353
Female	50(49.5)	300.8 ± 187.5		188.6 ± 70.5		37.7 ± 16.1		24.0 ± 11.1	
Age (years)									
≥ 60	45(44.6)	303.4 ± 187.7	0.562	203.5 ± 87.0	0.696	36.9 ± 16.4	0.159	22.7 ± 11.2	0.019*
< 60	56(55.4)	298.1 ± 133.9		192.7 ± 73.9		41.6 ± 18.2		27.1 ± 11.4	
Tumor size (cm)									
≥ 5	29(28.7)	358.8 ± 183.5	0.029*	198.9 ± 63.9	0.684	43.0 ± 14.9	0.071	26.5 ± 8.8	0.165
< 5	72(71.3)	277.0 ± 143.2		196.9 ± 85.8		38.1 ± 18.3		24.6 ± 12.4	
Differentiation									
Well	16(15.8)	296.4 ± 158.4	0.078	200.9 ± 86.1	0.976	39.7 ± 17.4	0.601	26.7 ± 13.1	0.891
Moderate	35(34.7)	353.8 ± 187.5		196.0 ± 69.2		37.9 ± 18.8		25.4 ± 12.1	
Poor	50(49.5)	264.5 ± 127.9		197.5 ± 86.0		40.6 ± 16.1		24.5 ± 10.6	
Lymph node metastasis									
Positive	66(65.3)	329.1 ± 172.3	0.014*	210.8 ± 80.7	0.019*	40.1 ± 16.7	0.438	27.9 ± 11.2	0.0002*
Negative	35(34.7)	246.5 ± 115.4		172.4 ± 72.7		38.5 ± 19.0		20.0 ± 10.1	
Distant metastasis									
Positive	18(21.7)	304.1 ± 122.3	0.435	218.8 ± 91.4	0.371	41.4 ± 15.1	0.297	25.6 ± 10.5	0.572
Negative	83(78.3)	299.7 ± 166.8		192.9 ± 76.9		39.1 ± 18.0		25.1 ± 11.7	
TNM stage									
I	28(27.7)	234 ± 119.7	0.0062*	175.8 ± 78.3	0.0571	37 ± 19.4	0.7371	19.3 ± 9	0.0016*
II	13(12.9)	241.8 ± 101.5		182.5 ± 84.6		40.9 ± 15.6		21.7 ± 10.9	
III	36(35.6)	329.5 ± 173.2		195.9 ± 66.1		39.2 ± 17.7		27.8 ± 11.1	
IV	24(23.8)	366.2 ± 171.2		233.4 ± 89.7		42.3 ± 16.4		29.9 ± 12	
Healthy voluteers									
Gender									
Male	26(56.5)	142.8 ± 62.8	0.475	128 ± 56.6	0.784	29.2 ± 17.1	0.33	17.4 ± 10.6	0.123
Female	20(43.5)	172 ± 101.1		133.3 ± 63.2		25.1 ± 14.7		13.1 ± 7.3	
Age (years)									
≥ 60	18(39.1)	158.2 ± 67.2	0.632	146.7 ± 66.8	0.249	28.4 ± 21.4	0.486	16.4 ± 12.2	0.726
< 60	28(60.9)	153.7 ± 91.4		119.8 ± 51.8		26.8 ± 11.9		15 ± 7.4	

*means *P*-value< 0.05

### RNA isolation and reverse transcription

Total circulating miRNA was extracted from 200 μL plasma using the miRNeasy Serum/Plasma Kit (Qiagen) according to the manufacturer’s protocol. In addition, 10 μL of a 1.5 nmol/L solution of the custom synthetic miRNA cel-miR-54-5p was added after the sample was mixed with 1 mL QIAzol Lysis reagent for 5 min. RNA was eluted from spin columns in 40 μL nuclease-free water.

Four circulating human miRNAs (miR-21, miR-93, miR-106a and miR-106b) and one spike-in control miRNA (cel-miR-54-5p) were determinated by TaqMan™ MicroRNA Assays, TaqMan miRNA Reverse Transcription kits (Life Technologies) and miRNA-specific RT primers were used for reverse transcription. For each sample, 3 μL RNA sample was added in a 15 μL reaction mixture using standard protocol. Then, the resulting cDNA was prepared for the droplet digital PCR.

### ddPCR workflow

For each ddPCR assay, 3 μL cDNA sample, 10 μL 2× ddPCR supermix for probes (Bio-Rad), 1 μL 20× TaqMan miRNA probe and 6 μL RNase-free Water was added in a 20 μL reaction mixture. Then, the mixture and 70 μL droplet generation oil for probes (Bio-Rad) were respectively loaded into the sample wells and oil wells of a disposable droplet generator cartridge (Bio-Rad). After that, droplets were generated by QX200 droplet generator device (Bio-Rad) and carefully transferred to a 96-well PCR plate (Eppendorf). The cycling conditions were: 95 °C for 10 min, 40 cycles of 95 °C for 15 s and 57 °C for 1 min, and a final step at 98 °C for 10 min. At the end of the PCR reaction, droplets were read in the QX200 droplet reader and analyzed using the Quantasoft™ version 1.7.4 software (Bio-Rad). In addition, a no template control (NTC) was included in every assay. And the spike-in control miRNA was used as an internal calibrator to monitor extraction efficiency.

### Statistical analysis

The statistical analyses were performed using the SPSS version 19.0 software. The Mann-Whitney U test was used to compare significant differences in miRNA expression between different groups. Logistic regression was used to develop a combined miRNA panel to diagnose GC with different TNM stage. Receiver operating characteristic (ROC) curves were established to evaluate the capacity of the tested miRNA to discriminate cancer cases in different TNM stage, and its potential use as a diagnostic tool for detecting GC. A *p*-value of less than 0.05 was considered to be significant.

A total of 147 participants in the training cohort were grouped into the training data set, and 28 participants in the validation cohort were grouped into the testing data set. In the training stage, a classical random forest algorithm in R version 3.4.2 software was used to construct variable selection models for combined four miRNA panel and clinical parameters in this study. Next, using single blind method, we tested the model by using the 28 cases of the testing data set as a prospective validation set, to assess its predictive ability. And we also retrospectively analyzed the 147 cases of the training data set.

## Results

### Circulating miRNAs in plasma of GC patients versus healthy controls

**Fig. 1:**
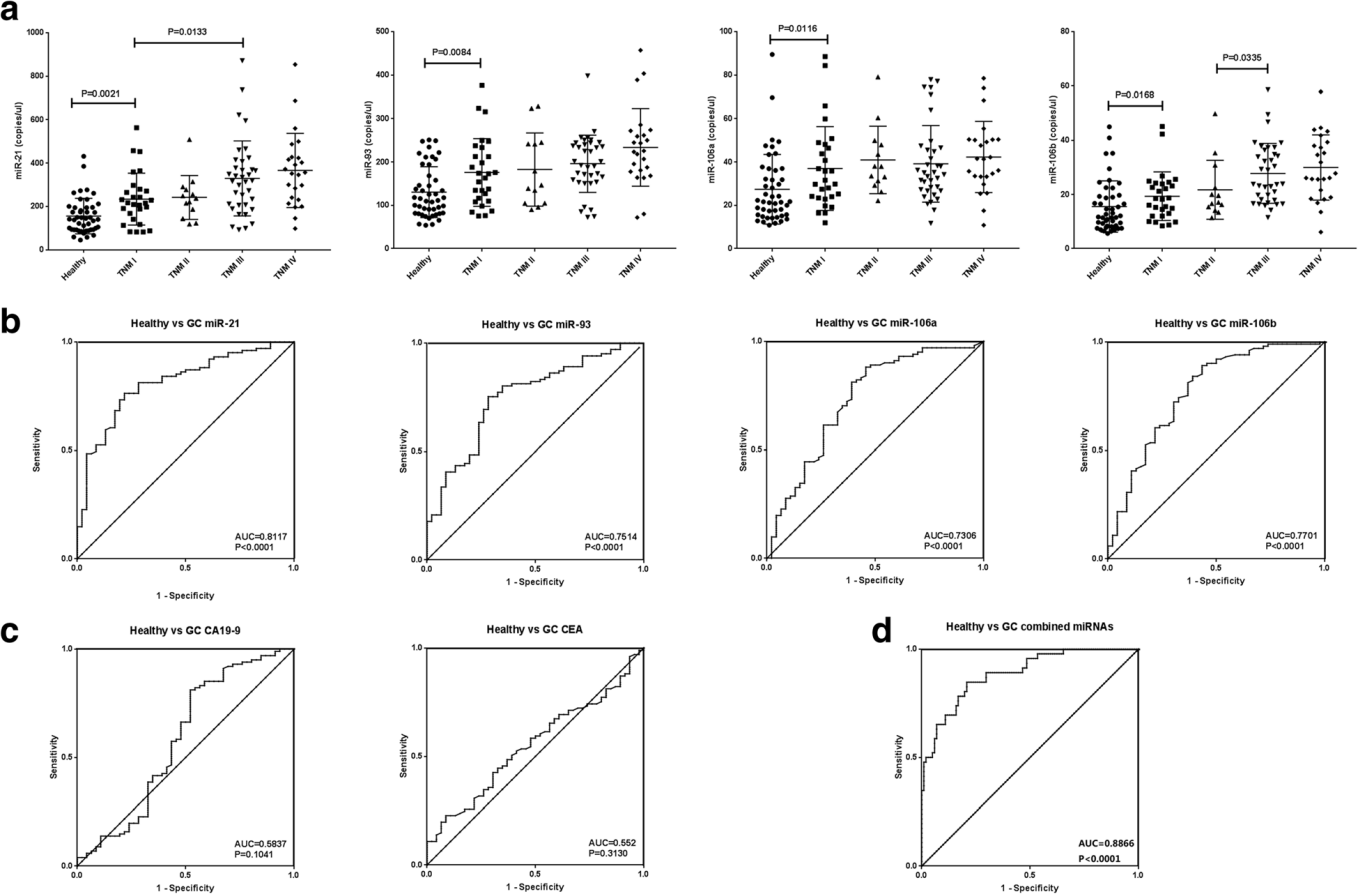
Diagnostic value of circulating miRNAs expression signature in discriminating gastric cancer patients from healthy volunteers. **a** Levels of circulating miR-21, miR-93, miR-106a and miR-106b in plasma of gastric cancer patients and healthy volunteers. The levels of miRNA are presented as copies/μl of PCR reaction. **b** ROC analysis for individual miRNA. **c** ROC analysis for the common tumor biomarkers including CEA and CA19–9. **d** ROC analysis for the combined miRNA panel

First, we compared the expression levels of four validated miRNAs in plasma from healthy volunteers (*n* = 46) and GC patients (*n* = 101) with different TNM stage using ddPCR. All four miRNAs including miR-21, miR-93, miR-106a and miR-106b levels were significantly lower in healthy controls than GC patients with TNM stage I (*p* = 0.0021, *p* = 0.0084, *p* = 0.0116 and *p* = 0.0168 respectively) (Fig. 1a), as well as TNM stage II, III and IV (Table 2). To evaluate the diagnostic value of the concentrations of these four circulating miRNAs, ROC curve analysis was performed. GC patients with different TNM stage were combined as one group, the area under the curve (AUC) values of miR-21, miR-93, miR-106a and miR-106b were 0.811 (95% confidence interval [CI], 0.739–0.884), 0.751 (95% CI, 0.667–0.836), 0.731 (95% CI, 0.638–0.823) and 0.77 (95% CI, 0.683–0.857), respectively (Fig. 1b). We also detect the CEA and CA19–9 in all 147 participants in the present study, the testing time was pre-surgery for GC patients. The AUC values obtained for CEA and CA19–9 to distinguish the GC patients from the healthy controls were 0.552 (95% CI, 0.456–0.648) and 0.584 (95% CI, 0.473–0.695), respectively (Fig. 1c).

**Table 2:** Performance of circulating miRNAs for detection of GC with different TNM stages

	miR-21	miR-93	miR-106a	miR-106b
	P-value			
Health vs GC TNM I	0.0021^**^	0.0084^**^	0.0116^*^	0.0168^*^
Health vs GC TNM II	0.0014^**^	0.0263^*^	0.0013^**^	0.0132^*^
Health vs GC TNM III	< 0.0001^**^	< 0.0001^**^	0.0004^**^	< 0.0001^**^
Health vs GC TNM IV	< 0.0001^**^	< 0.0001^**^	0.0001^**^	< 0.0001^**^
GC TNM I VS II	0.5704	0.8142	0.2820	0.6043
GC TNM I VS III	0.0133^*^	0.1543	0.4864	0.0018^**^
GC TNM I VS IV	0.0018^**^	0.0102^*^	0.1118	0.0004^**^
GC TNM II VS III	0.0791	0.4665	0.5262	0.0335^*^
GC TNM II VS IV	0.0163^*^	0.0952	0.6097	0.0200^*^
GC TNM III VS IV	0.2985	0.0897	0.2164	0.3092

* means *P* < 0.05, ** means *P* < 0.01

Furthermore, through a combination of the expression levels of four validated miRNAs, weighted by the regression coefficient, we developed a miRNA classifier using logistic regression model. It could be used to evaluate the predicted probability of being detected as GC, which was calculated as follows: first, the expression levels of four miRNAs were calculated as miRNA panel score using the following equation: miRNA panel score = 5.218–0.011 × miR-21-0.012 × miR-93-0.037 × miR-106a-0.031 × miR-106b. Then, the predicted probability was calculated by a second equation: predicted probability = EXP (miRNA panel score)/[1 + EXP (miRNA panel score)]. The combination of the four miRNAs exhibited better diagnostic value compared to any individual miRNA, with an AUC of 0.887 (95% CI, 0.83–0.943) (Fig. 1d) by logistic regression analysis, an optimal cut-off point was indicated at 0.315 with a sensitivity of 84.8% and a specificity of 79.2%. These results indicated that the circulating miR-21, miR-93, miR-106a and miR-106b could be considered as more accurate biomarkers than CEA and CA19–9 for GC diagnosis.

### Circulating miRNAs in plasma of GC patients with different TNM stage

Although four miRNAs were up-regulated in GC patients with TNM stage II compared with stage I, as well as TNM stage IV compared with stage III, but the differences had no statistically significant (*p* > 0.05) (Fig. 1a & Table 2). However, miR-21 and miR-106b levels were significantly increased in GC patients with TNM stage III or IV compared with stage I (miR-21:*p* = 0.0133 and *p* = 0.0018, miR-106b:*p* = 0.0018 and *p* = 0.0004 respectively) (Table 2). miR-106b levels were still significantly increased when compared TNM stage III or IV with stage II in GC patients (*p* = 0.0335 and *p* = 0.02 respectively), while it was significant for miR-21 levels only when compared TNM stage IV with stage I in GC patients (*p* = 0.0163). Moreover, miR-93 and miR-106a levels in GC patients had no significant difference between groups with different TNM stage, except for the miR-93 levels in GC patients with TNM stage I and IV (*p* = 0.0102).

**Fig. 2:**
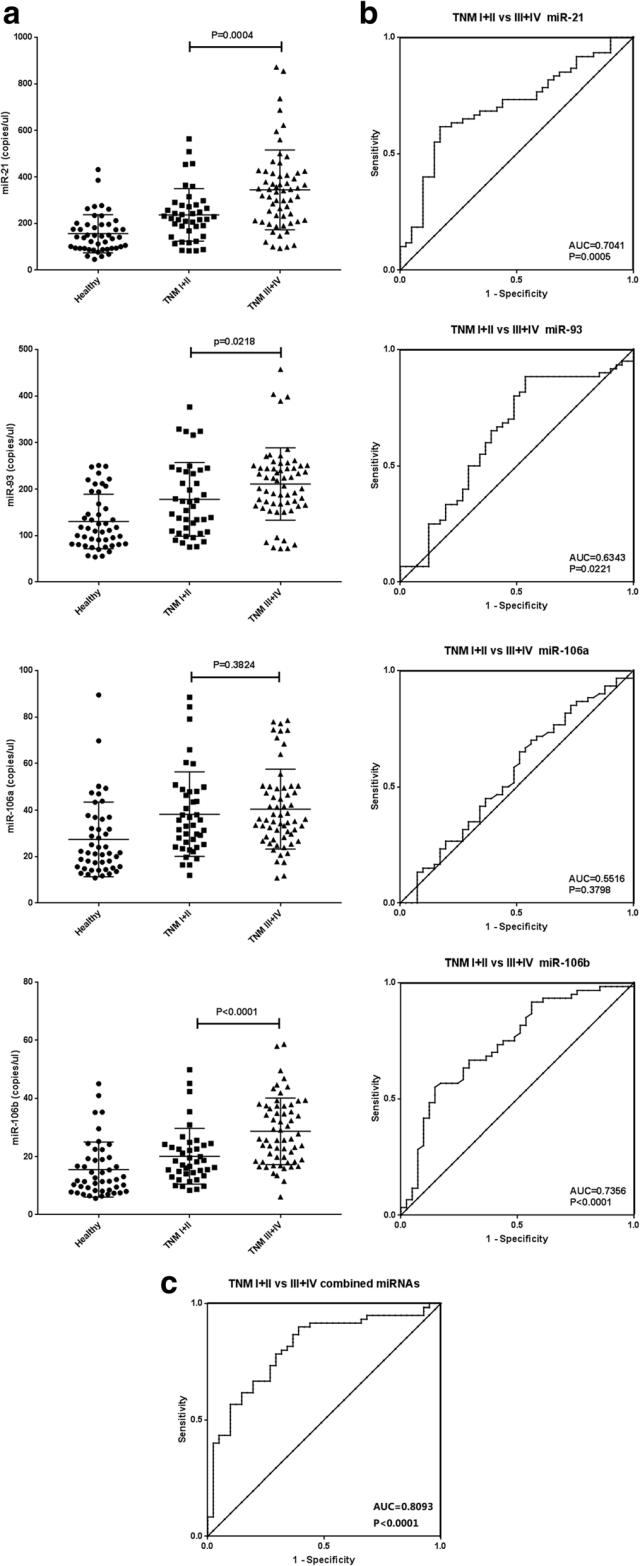
Diagnostic value of circulating miRNAs expression signature in discriminating gastric cancer at different TNM stage. **a** Levels of circulating miR-21, miR-93, miR-106a and miR-106b in plasma of gastric cancer patients with low TNM stage (stage I and II) and high TNM stage (stage III and IV), and healthy volunteers. The levels of miRNA are presented as copies/μl of PCR reaction. **b** ROC analysis for individual miRNA. **c** ROC analysis for the combined miRNA panel

Furthermore, we combined GC patients with TNM stage I and II as one group, as well as TNM stage III and IV. As expected, the results showed that miR-21 and miR-106b levels were significantly higher in stage III and IV compared to stage I and II (*p* = 0.0004 and *p* < 0.0001 respectively), while miR-106a levels still had no significant difference between these two groups (*p* = 0.3824) (Fig. 2a). However, there was an unexpected significant increase for miR-93 levels in GC patients with stage III and IV compared to stage I and II (*p* = 0.0218) (Fig. 2a). The AUC values obtained for miR-21, miR-93, miR-106a and miR-106b to distinguish the GC patients with stage I and II from stage III and IV were 0.704 (95% CI, 0.601–0.807), 0.634 (95% CI, 0.52–0.749), 0.552 (95% CI, 0.435–0.668) and 0.736 (95% CI, 0.635–0.836), respectively (Fig. 2b).

Same as previous analysis, we assigned each patient a risk score which was calculated as follows: First, miRNA panel score = − 2.875 + 0.005 × miR-21 + 0.003 × miR-93-0.034 × miR-106a + 0.115 × miR-106b. Then, risk score = EXP (miRNA panel score)/[1 + EXP (miRNA panel score)]. The combination of the four miRNAs exhibited better capability to discriminate GC with TNM stage I and II from stage III and IV compared to any individual miRNA, with an AUC of 0.809 (95% CI, 0.723–0.896) (Fig. 2c) by logistic regression analysis, an optimal cut-off point was indicated at 0.534 with a sensitivity of 78.3% and a specificity of 70.7%. Taken together, these results demonstrated that circulating miR-21, miR-93 and miR-106b might have a potential diagnostic value for distinguishing GC with different TNM stage.

### Correlation between expression levels of circulating miRNA in plasma and clinicopathologic factors in GC patients

Except for the TNM stage, we evaluated whether the levels of four circulating miRNAs are correlated with other clinical characteristics of all GC patients. As it was summarized in Table 1, the expression of miR-21, miR-93, miR-106a and miR-106b didn’t significantly differ between the GC patients based on gender (*p* = 0.437, *p* = 0.318, *p* = 0.404 and *p* = 0.353 respectively), tumor differentiation (*p* = 0.078, *p* = 0.976, *p* = 0.601 and *p* = 0.891 respectively) and distant metastasis (*p* = 0.435, *p* = 0.371, *p* = 0.297 and *p* = 0.572 respectively). However, the levels of miR-21, miR-93 and miR-106b in plasma from the GC patients is significantly related to lymph node metastasis (*p* = 0.014, *p* = 0.019 and *p* = 0.0002 respectively). Moreover, the circulating miR-21 and miR-106b levels are also related to tumor size and age respectively (*p* = 0.029 and *p* = 0.019). The results demonstrated the feasibility of these miRNAs for the diagnosis of other clinicopathological characteristics of GC patients.

### Random forest model used for GC diagnosis

To evaluate the diagnostic value of circulating miR-21, miR-93, miR-106a and miR-106b combined with other conventional clinical parameters including gender, age, CEA and CA19–9 for GC with different TNM stage, we used a classical random forest algorithm for analysis. A total of 147 participants in the training cohort were grouped as the training data set and used for developing the model. And the other 28 participants in the validation cohort were grouped as the testing data set and used for assessing predictive ability of the model.

In the training stage, using random forest supervised classification algorithm, four microRNAs, three clinical parameters including CEA, age and CA19–9 mostly related to the diagnostic classification were selected (Additional file 1: Figure S1). In the testing stage, we used the developed random forest model to validate both the training data set and the testing data set. It correctly discriminated 147 out of 147 samples in the training data set, and 23 out of 28 samples in the testing data set, which showed 100 and 82.1% accuracy respectively in identifying Healthy volunteers and GC patients with different TNM stage (Table 3), by using the selected variables based on their value cut-offs (Additional file 1: Figure S1). In addition, the most influential factor in this model was miR-21, followed by miR-106b, miR-93, miR-106a, CA19–9, CEA, age and gender (Table 4).

**Table 3:** Confusion matrix of the developed random forest model in the testing stage

		Predicted class		
		Healthy	TNM I + II	TNM III + IV
Training data set				
Actual class	Healthy	46	0	0
	TNM I + II	0	41	0
	TNM III + IV	0	0	60
Testing data set				
Actual class	Healthy	14	2	1
	TNM I + II	0	4	1
	TNM III + IV	1	0	5

**Table 4:** The relative importance of variables in the developed random forest model

Variables	Mean Decrease Gini
Gender	1.134787
Age	9.342937
CA19–9	11.051689
CEA	9.918123
miR-21	19.143754
miR-93	16.274413
miR-106a	12.627928
miR-106b	16.550531

## Discussion

Early diagnosis could greatly improve the survival rates of GC patients. However, the currently used diagnostic methods are either invasive or insensitive, thus limited their application in clinic. In recent years, a number of circulating miRNAs, which are notably stable in the circulation of body fluids [20, 21], are suggested as promising non-invasive diagnostic markers for GC [9, 15, 22–24]. Unfortunately, since circulating miRNAs exist in blood at extremely low concentrations [25], the test results would be made poorly repeatable due to the interference of several variables, such as sample processing protocols, RNA isolation and so on [10, 26]. Most importantly, quantitative real-time PCR is most commonly used but must rely on the use of external calibrators, because it lacks reliable endogenous reference miRNA for normalization of results in plasma or serum. Therefore, the data which produced by a variety of normalization methods in different studies, become non-comparable or difficult to compare. This is a major obstacle for their translation into clinically useful applications [10, 27].

The present study, to our best knowledge, is the first to evaluate the diagnostic value of circulating miRNAs for GC patients using the ddPCR technique. ddPCR is a recently introduced technology which can achieve absolute quantification of nucleic acids based on the principles of sample portioning, end-point PCR and Poisson statistics [28, 29]. Thus, it overcomes the normalization and calibrator issues [30]. Besides, it has shown better precision and sensitivity while detecting low concentration of target nucleic acids molecules [31, 32]. More importantly, ddPCR can tolerate PCR inhibitors which could influence the efficiency of PCR amplification, without affecting the quantitative results of the target [11].

Using ddPCR, we analyzed the levels of circulating miR-21, miR-93, miR-106a and miR-106b in the plasma of GC patients and healthy volunteers. Similar to previous studies [9, 14, 15], we found the significantly increased levels of these miRNAs in GC patients compared with healthy controls, and some miRNAs were associated with advanced TNM stage. ROC curve analysis showed that each miRNA had higher diagnostic sensitivity and specificity than CEA and CA19–9 which were widely used in clinic. Furthermore, through a combination of the expression levels of four validated miRNAs, a patient will be considered to have GC if the predicted probability is higher than the threshold set (0.315 with a sensitivity of 84.8% and specificity of 79.2%) in the model. An AUC of 0.887 (95% CI, 0.83–0.943) and *P*-values< 0.001 indicate the great potential value of these miRNAs as GC biomarkers.

Based on the results above, we further evaluate the potential use of these miRNAs in discriminating GC with different TNM stage. First, GC patients with TNM stage I and II were combined as one group, as well as stage III and IV, because there was no statistically significant difference between these groups. Then, our results showed that the levels of circulating miR-21, miR-93 and miR-106b in the plasma of GC patients were significantly higher in TNM stage III and IV than stage I and II, except for the miR-106a. As usual, a combination of four miRNAs showed better capability to discriminate GC with different TNM stage. A patient will be considered to have GC with TNM stage III or IV if the risk score is higher than 0.534 (a sensitivity of 78.3% and specificity of 70.7%). ROC analysis also showed an AUC of 0.809 (95% CI, 0.723–0.896) and P-values< 0.001. To our knowledge, this study is the first to demonstrate that these miRNAs might be also used as biomarkers to discriminate GC with TNM stage I and II from stage III and IV.

In the search of possible correlations with clinicopathological features, it was noteworthy that the presence of lymph node metastases was significantly correlated with increased levels of circulating miR-21, miR-93 and miR-106b. Moreover, a high level of circulating miR-21 was significantly related to a bigger tumor size (≥5 cm). These results indicate that these miRNAs might represent biomarkers of tumor aggressiveness, which further improved their value for discriminating GC with different TNM stage. Some studies have reported that high levels of miR-21 expression may induce tumor proliferation, migration and invasion via the downregulation of Noxa or PTEN expressions in GC cells [33, 34]. And miR-93 could promote proliferation and metastasis of GC via targeting TIMP2 or inactivation of the Hippo signaling pathway [35, 36]. In cancer-associated fibroblasts from GC, miR-106b could promote cell migration and invasion by targeting PTEN [37]. And it could also promote cell cycling of GC cells through regulation of p21 and E2F5 target gene expression [38]. These might be the mechanism of its correlation with lymph node metastases and tumor size. However, although it was reported that miR-106a could also regulate invasion and metastasis of GC via targeting TIMP2 [39, 40], and may inhibit extrinsic apoptotic pathway through targeting FAS [41], our results demonstrated that miR-106a expression was not associated with the lymph node metastases and tumor size. Further studies are required.

In clinic, due to the numerous factors that influence the precision and accuracy of diagnosing diseases or predicting of patients’ prognosis, more and more studies are applying machine learning algorithms to medical data, including the detection of GC [20, 42, 43]. There are several algorithms such as random forest, support vector machine and neural networks were commonly used [43, 44]. Here, we chose random forest model since it is easy to interpret, and allowed us to estimate the importance of a variable. After the random forest model was established in the training stage, when we tested the predictive value of this model using the testing data set, our results showed that it correctly discriminated 14 out of 17 healthy volunteers (false rate, 17.6%), 4 out of 5 GC patients with TNM stage I or II (false rate, 20%), and 5 out of 6 GC patients with TNM stage III or IV (false rate, 16.7%). However, the number of cases included in the present study is still far from sufficient to develop a reliable model, and we also didn’t have enough cases to test and validate the model. Further studies with much more cases are urgently required, to improve their application in clinic. Moreover, despite our results and accumulating evidences suggested that circulating miRNAs stably existed in circulation and can indeed be used as biomarkers to identify and monitor a variety of cancers and other diseases, it is still unknown how and why GC causes changes in the levels of these four circulating miRNAs, and whether or how they play roles in physiology. Further studies are also needed.

## Conclusions

Overall, the present study demonstrated that by using the ddPCR technique, circulating miR-21, miR-93, miR-106a and miR-106b could be used as diagnostic plasma biomarkers in gastric cancer patients.

## Additional files

Additional file 1: **Figure S1.** A random forest model for discriminating healthy volunteers, gastric cancer patients with low TNM stage (stage I and II) and high TNM stage (stage III and IV) G0 represents healthy volunteers; G1 represents GC patients with TNM stage I and II; G2 represents GC patients with TNM stage III and IV. (TIF 624 kb)

## Abbreviations

AFP: α-fetoprotein
AUC: Area under the curve.
CA19–9: Carbohydrate antigen 19–9
CEA: Carcinoembryonic antigen
ddPCR: Droplet digital PCR
GC: Gastric cancer
miRNA: Microrna
qPCR: Quantitative real-time PCR
ROC: Receiver operating characteristic
