# Supplementary figures and images for: Droplet digital PCR-based circulating microRNA detection serve as a promising diagnostic method for gastric cancer
Source: BMC Cancer. 2018 Jun 22;18:676. doi: 10.1186/s12885-018-4601-5 (PMC6013872; doi:10.1186/s12885-018-4601-5)

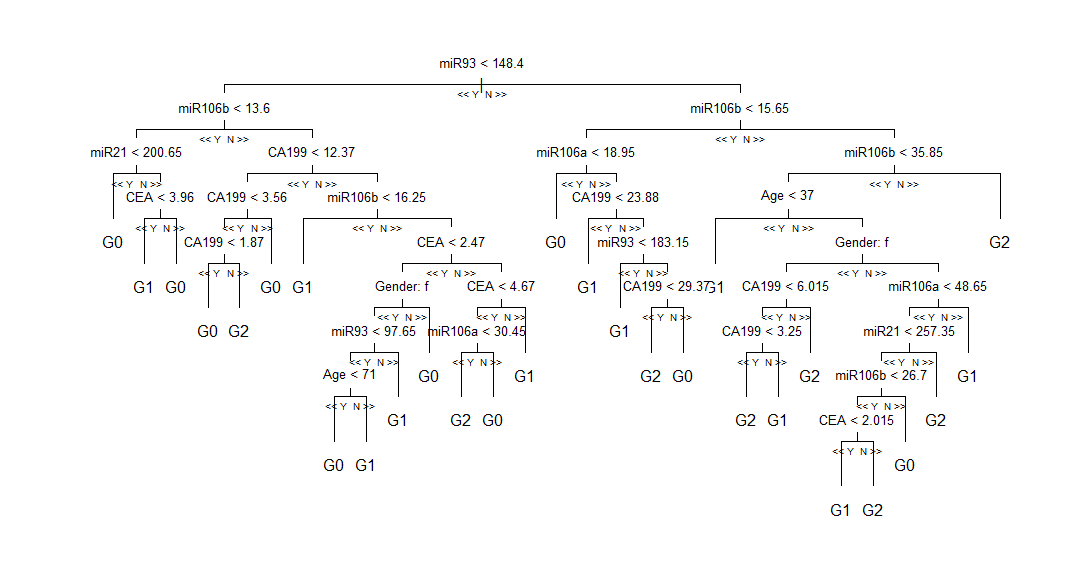

Supplement: Supplementary file 1 — Figure S1. A random forest model for discriminating healthy volunteers, gastric cancer patients with low TNM stage (stage I and II) and high TNM stage (stage III and IV) G0 represents healthy volunteers; G1 represents GC patients with TNM stage I and II; G2 represents GC patients with TNM stage III and IV. (TIF 624 kb) [file 12885_2018_4601_MOESM1_ESM.tif]
